# Left Atrial Appendage Occlusion in the Era of Minimalist Approaches: Anesthesia and Imaging Considerations

**DOI:** 10.3390/jcm15093396

**Published:** 2026-04-29

**Authors:** Giulia Laterra, Lorenzo Scalia, Orazio Strazzieri, Federica Agnello, Claudia Reddavid, Salvatore Ingala, Daniela Russo, Chiara Barbera, Simona Guarino, Giampiero Vizzari, Antonio Micari, Massimiliano Mulè, Marco Barbanti

**Affiliations:** 1 Faculty of Medicine and Surgery, Università Degli Studi di Enna “Kore”, Piazza dell’Università, 94100 Enna, Italy; federicagiuseppa.agnello@gmail.com (F.A.); massimiliano.mule@unikore.it (M.M.); mbarbanti83@gmail.com (M.B.); 2 Division of Cardiology, Ospedale Umberto I, ASP 4 di Enna, 94100 Enna, Italy; lorenzoscalia1993@gmail.com (L.S.); o.strazzieri@gmail.com (O.S.); claudiareddavid@gmail.com (C.R.); ingalasalvatore@gmail.com (S.I.); danielarusso28@hotmail.it (D.R.); chiara.barbera.me@gmail.com (C.B.); simonaguarino@hotmail.it (S.G.); 3 Department and Clinical and Experimental Medicine, “G. Martino” University Hospital, Via Consolare Valeria 1, 98124 Messina, Italy; giampierovizzari@hotmail.it (G.V.); micariantonio@gmail.com (A.M.); 4 Cardiology Unit, IRCCS, Istituto Mediterraneo Trapianti e Terapie ad Alta Specializzazione (ISMETT), Via Ernesto Tricomi, 5, 90127 Palermo, Italy

**Keywords:** left atrial appendage occlusion, intraprocedural imaging, atrial fibrillation, transesophageal echocardiography, intracardiac echocardiography, minimalist approach

## Abstract

The progressive aging of the atrial fibrillation (AF) population, frequently characterized by high ischemic and bleeding risks, has led to a substantial increase in referrals for left atrial appendage occlusion (LAAO). The expansion of indications and the high procedural success rate of LAAO have further contributed to rising procedural volumes. However, this growth introduces important challenges: LAAO candidates are often elderly and frail, with increased anesthesia-related risks, and high-volume catheterization laboratories may face logistical constraints, particularly in centers without dedicated anesthesiology support. The current gold standard approach, transesophageal echocardiography (TEE) under general anesthesia (GA), ensures optimal imaging and procedural control but may increase procedural complexity and perioperative risks. In response, minimalist strategies are increasingly explored, targeting either the anesthetic protocol or the imaging modality. Conscious sedation (CS) protocols have been adopted to reduce anesthesia-related burden while maintaining TEE guidance. Alternatively, imaging-based strategies aim to replace TEE with less invasive modalities, including intracardiac echocardiography (ICE), transesophageal–intracardiac echocardiography (TE-ICE), and MicroTEE. Each approach presents specific advantages and limitations regarding safety, feasibility, operator expertise, and institutional resources. Taken together, these findings support a patient-centered approach to LAAO, whether traditional or minimalist, in which the choice of anesthetic strategy and echocardiographic guidance is driven by institutional resources, operator expertise, and individual patient characteristics rather than by expected differences in procedural or clinical efficacy. This review summarizes current evidence on minimalist LAAO pathways and discusses their role in achieving a tailored, resource-conscious procedural model.

## 1. Introduction

Atrial fibrillation (AF) is the most prevalent cardiac arrhythmia worldwide, with a burden that is projected to increase with population aging. AF accounts for approximately 20–30% of ischemic strokes [1,2,3]. In patients with non-valvular AF, nearly 90% of cardioembolic strokes originate from thrombus formation within the left atrial appendage (LAA). This pathophysiological evidence represents the rationale for left atrial appendage occlusion (LAAO) as an alternative to oral anticoagulation (OAC) in selected patients. The 2019 AHA/ACC/HRS guidelines and the 2020 ESC guidelines for the diagnosis and management of AF recommend LAAO for stroke prevention in patients with non-valvular AF who have relative or absolute contraindications to long-term anticoagulation (Class IIb) [1,4,5,6]. Currently, most LAAO procedures are performed under transesophageal echocardiographic (TEE) guidance and general anesthesia (GA) [7]. However, frailty and multiple comorbidities frequently characterize patients referred for LAAO, raising concerns regarding the routine use of GA and TEE in this vulnerable population. In addition, the logistical complexity associated with coordinating multiple specialists represents a significant challenge to catheterization laboratory workflow efficiency [7]. These considerations have prompted increasing interest in minimalist procedural strategies aimed at avoiding general anesthesia while maintaining procedural safety and efficacy. In this context, a “minimalist approach” refers to a procedural strategy aimed at reducing the use of general anesthesia, while simultaneously simplifying procedural logistics by minimizing the need for coordination among multiple specialist teams. In this framework, same-day discharge should be considered a potential downstream consequence of a streamlined and less invasive approach, rather than a defining feature of the strategy itself. Minimalist strategies are increasingly explored, targeting either the anesthetic protocol or the imaging modality. This manuscript was conducted as a narrative review of the available literature. A literature search was performed using PubMed/MEDLINE and Scopus databases, using combinations of the following keywords: “left atrial appendage occlusion”, “LAAO”, “transesophageal echocardiography”, “intracardiac echocardiography”, “minimalist approach”, “conscious sedation”, and “general anesthesia”. Articles published in English over the last 10–15 years were preferentially considered, with particular emphasis on prospective studies, comparative analyses, and high-quality observational data. Study selection was based on clinical relevance and methodological quality, with the aim of providing a comprehensive and clinically oriented overview rather than a formal systematic synthesis of the evidence. Additional relevant studies were identified through manual screening of reference lists.

## 2. Standard Approach: TEE-Guided LAAO Under General Anesthesia

All expert consensus documents recommend the use of intraprocedural imaging, either TEE or intracardiac echocardiography (ICE), to guide LAAO procedures [8]. This recommendation is based on evidence showing that intraprocedural echocardiography, compared with fluoroscopy alone, is associated with lower procedural complication rates and higher long-term technical success [9]. In centers using fluoroscopy alone, LAA sizing is performed angiographically after transseptal puncture, with measurements comparable to those obtained by TEE [10]. Although different imaging strategies have been described (TEE, ICE, or fluoroscopy alone), the combination of fluoroscopy and TEE remains the most widely adopted approach in clinical practice. Imaging is essential throughout the different procedural steps of LAAO. First, it is used to rule out the presence of LAA thrombus. Second, it guides transseptal puncture (TSP), enabling a safer puncture and facilitating coaxial access to the LAA. TEE plays a crucial role in achieving optimal TSP. The inferior and posterior positions are typically determined using the long-axis 110° view of the interatrial septum and the short-axis 30° view of the septum, respectively. For the Watchman device (Boston Scientific, Marlborough, MA, USA) and the Amplatzer Amulet Cardiac Plug (Abbott Structural Heart, Plymouth, MN, USA), an inferior–posterior transseptal puncture is recommended. In contrast, for the LARIAT device (SentreHEART Inc., Redwood City, CA, USA), a superior–posterior transseptal puncture is suggested. Third, it assists in device positioning, which is typically evaluated using TEE in multiple views (0°, 45°, 90°, and 135°). For the different closure devices, specific imaging criteria have been proposed to confirm optimal device positioning, stability, and adequate occlusion before final release. For the Watchman FLX device, the PASS criteria are commonly used:

- Position: adequate shape and positioning of the device within the LAA;
- Anchor: satisfactory anchoring confirmed with a tug test to ensure device stability;
- Size/Compression: appropriate device compression (typically 10–30%);
- Seal: adequate closure of the LAA without significant peri-device leak.

For the Amulet device, the CLOSE criteria are applied:

- Closure: approximately two-thirds of the device lobe should be distal to the left circumflex artery;
- Lobe compression: adequate compression of the device lobe;
- Orientation: the device lobe should be aligned with the axis of the LAA neck;
- Separation: adequate separation between the lobe and the disc, with the disc assuming an elliptical configuration [11].

Fourth, it allows assessment of potential complications, such as pericardial effusion, evaluation of surrounding cardiac structures, and exclusion of peri-device leak or device embolization [11]. However, although TEE is generally considered a safe imaging modality, a number of complications related to probe insertion and manipulation have been reported. These complications are usually minor and transient, including sore throat, dysphagia, or minor mucosal injury. More severe complications, although rare, may occur and include esophageal perforation, upper gastrointestinal bleeding, hematoma, and, in exceptional cases, mediastinitis. The reported incidence of major complications is low, generally estimated to be below 0.5%, but the risk may increase in patients with pre-existing esophageal disease, prolonged procedures, or repeated probe manipulation [12]. In the setting of structural heart interventions, additional procedural factors may contribute to the risk of TEE-related complications. These include prolonged imaging times and continuous probe manipulation to obtain optimal views during device deployment [12,13]. GA may further increase this risk compared with conscious sedation, as patients lose protective physiological mechanisms such as spontaneous swallowing and normal esophageal peristalsis. These mechanisms are believed to facilitate physiological cooling and reduce mechanical stress on the esophageal wall. Consistent with these observations, Di Biase et al. reported that patients undergoing AF ablation under GA had a higher incidence of esophageal injury detected by capsule endoscopy compared with those treated under conscious sedation [14]. The authors suggested that the abolition of spontaneous deglutition and reduced esophageal peristalsis may impair physiological cooling mechanisms, thereby increasing susceptibility to esophageal injury. Despite these considerations, most TEE-related complications reported in the literature are self-limited and managed conservatively. Nevertheless, careful probe manipulation, awareness of patient-related risk factors, and minimizing unnecessary probe movement remain important strategies to reduce the risk of esophageal injury during structural heart interventions [12].

## 3. Alternative Anesthetic Strategies for TEE-Guided LAAO

LAAO procedures have traditionally been performed under GA, particularly in the United States, largely due to the need for TEE guidance. GA offers several advantages, including complete airway control, patient immobility, operator comfort, reliable analgesia and amnesia, as well as the ability to perform breath-holding maneuvers when needed. These features may facilitate procedural workflow and imaging quality. However, GA is also associated with increased procedural complexity, longer preparation and recovery times, higher resource utilization, and increased costs. MCS and deep sedation (DS) have emerged as feasible alternatives in selected patients. MCS is characterized by a depressed level of consciousness in which patients respond purposefully to verbal commands or light tactile stimulation while maintaining spontaneous ventilation and airway reflexes. In contrast, DS is associated with a deeper reduction in consciousness, where patients respond only to repeated or painful stimuli and may require assistance in maintaining airway patency and adequate ventilation [15,16,17,18,19]. As demonstrated in other structural heart interventions such as transcatheter aortic valve replacement (TAVR), less invasive anesthetic strategies can maintain procedural safety while reducing logistical burden. When appropriately administered, MCS may provide sufficient TEE tolerance and suppress agitation that could otherwise compromise procedural success. Transitioning from GA to MCS requires structured team training involving catheterization laboratory nurses, imaging cardiologists, anesthesiologists (when applicable), and interventionalists. The primary goal is to ensure patient comfort and safety while maintaining spontaneous breathing and minimizing movement that could interfere with device deployment. Several sedation protocols have been proposed to facilitate TEE-guided LAAO under MCS. Most strategies combine short-acting sedative agents with topical pharyngeal anesthesia to improve patient tolerance to TEE while maintaining spontaneous ventilation. Ates et al. described a moderate conscious sedation protocol including preprocedural glycopyrrolate and acetaminophen, with local anesthesia of the groin performed using prilocaine prior to vascular access. Midazolam and/or fentanyl were administered for anxiolysis and sedation when required, and a topical anesthetic spray was applied before TEE insertion to facilitate probe placement. Device deployment was not significantly affected by respiratory motion under MCS, and positioning stability was considered comparable to procedures performed under GA, indicating that the use of MCS did not compromise procedural success [20]. In a different approach, Vizzari et al. reported the use of dexmedetomidine combined with midazolam for sedation during TEE-guided procedures. Dexmedetomidine was administered as a continuous infusion titrated according to patient frailty and hemodynamic status, while midazolam facilitated probe insertion. Topical lidocaine spray was applied before TEE, and continuous monitoring of vital signs was maintained throughout the procedure. This strategy resulted in adequate procedural sedation with good hemodynamic and respiratory stability and high operator and patient satisfaction [21,22]. Marmagkiolis et al. evaluated the feasibility and safety of TEE-guided LAAO under MCS in a prospective study including 112 patients. Sedation was mainly achieved with a propofol bolus followed by continuous infusion, with adjunctive midazolam and fentanyl administered when required, and a topical anesthetic spray applied prior to TEE insertion. The procedure was successfully completed in all patients without conversion to GA and without MCS-related complications, allowing same-day discharge in selected patients [23]. Additional observational studies have further supported the feasibility and safety of conscious sedation during LAAO, demonstrating comparable procedural success and short-term outcomes across different patient risk profiles [24,25,26,27,28,29]. Currently, no standardized pharmacological protocol for conscious sedation in LAAO has been established, and most available data derive from observational studies using heterogeneous combinations of sedative and analgesic agents (Table 1). As a result, the choice of medications is largely driven by institutional protocols, operator experience, and patient characteristics, rather than by evidence-based comparisons.

## 4. Alternative Imaging Modalities for LAAO Guidance

### 4.1. Intracardiac Echocardiography (ICE)

ICE has been used for several decades to guide structural heart interventions and electrophysiological procedures, including atrial septal defect and patent foramen ovale closure, as well as transseptal puncture [36,37,38]. In many centers, ICE has become the default imaging modality for LAAO guidance, as it allows procedures to be performed under local anesthesia with the patient awake and responsive, avoiding the need for GA. ICE systems have evolved from early rotational catheters, which are limited by the lack of steerability and near-field imaging, to current phased-array catheters that provide steerability, Doppler capabilities, and far-field imaging, making them more suitable for structural heart disease interventions [39]. In the context of LAAO, ICE can be used from different intracardiac locations, including the right atrium, right ventricle, pulmonary artery, or via transseptal access within the left atrium. Although imaging from right-sided structures is feasible, positioning the catheter in the left atrium generally provides more accurate and reproducible visualization of the LAA, facilitating device sizing and deployment. The ICE LAAC study is the first prospective study of ICE-guided LAAC with independent adjudication of imaging and clinical outcomes, showing high procedural success, low complication rates, and effective LAA closure with the Watchman FLX device [30]. Several studies have compared TEE- and ICE-guided LAAO, showing that ICE-guided procedures are associated with similar clinical outcomes, hospital costs, and no differences in procedural or vascular complications compared with TEE-guided LAAO, across different LAAO devices [31,32,33,40]. A key benefit of ICE is the simplification of procedural logistics, as the operator can independently control both the imaging modality and interventional devices. This approach may eliminate the need for general anesthesia and for a dedicated echocardiographer to perform TEE, thereby streamlining workflow. Despite the established advantage of avoiding GA, the use of ICE has several limitations. These include the need for additional vascular access with potential procedural risks, the lack of standardized imaging protocols, and the technical complexity associated with visualizing the highly variable anatomy of the LAA [39]. In addition, image quality may be suboptimal from certain positions, often requiring multiple catheter manipulations or left atrial access to achieve adequate visualization. However, Berti et al. reported that although ICE may provide lower image quality compared with TEE, this does not appear to impact technical success [32]. Given the intrinsic limitations of ICE in accurately sizing the LAA, preprocedural imaging (computed tomography or TEE) remains essential. Nevertheless, this limitation may be mitigated by the advent of 3D ICE probes, which have already been investigated for guiding LAAO, demonstrating a high level of agreement in LAA sizing compared with preprocedural TEE [41]. Furthermore, ICE-guided LAAO is associated with a learning curve, which may be shortened through structured operator training, including dedicated courses and workshops, simulator-based training, and live case demonstrations [32] (Table 1).

### 4.2. Miniaturized TEE Probes

The three main types of transesophageal echocardiography probes currently used in structural heart interventions differ substantially in terms of size, imaging capabilities, and transducer technology. The standard TEE probe has a shaft diameter of approximately 10 mm and a tip size of about 16 × 12 mm. This is the most used probe for intraprocedural imaging. In contrast, the mini-TEE probe has a smaller shaft diameter of approximately 7.4 mm and a tip size of 10.7 × 7.2 mm, while the micro-TEE probe is even more compact, with a shaft diameter of about 5.2 mm and a tip size of approximately 7.5 × 5.5 mm. Another important difference concerns the number of transducer elements. Standard TEE probes contain more than 2500 elements, enabling high-resolution imaging. In contrast, mini-TEE probes include approximately 48 elements and micro-TEE probes about 32 elements, reflecting a simplified transducer architecture. These technological differences translate into distinct imaging capabilities. Standard TEE probes allow both two-dimensional (2D) and real-time three-dimensional (3D) imaging. By comparison, mini-TEE and micro-TEE probes currently provide 2D imaging only. Despite this limitation, their markedly reduced size may improve patient tolerance and facilitate the use of MCS or minimalist procedural strategies [28]. Patti et al. reported their experience using micro-TEE guidance for LAAO performed under local anesthesia and conscious sedation with intravenous midazolam and fentanyl. In the initial phase, the first five procedures were attempted using a transnasal approach; however, mild epistaxis occurred in two patients, prompting the investigators to subsequently adopt a transoral approach for the remaining procedures. Overall, micro-TEE guidance was well tolerated throughout the procedure without airway complications. Image quality was assessed by two independent echocardiographers and was rated as good in 73.5% of cases and acceptable in 26.5% [25]. Aminian et al. reported the largest multicenter experience to date with the use of miniaturized TEE probes for LAAO, including 546 consecutive procedures performed in five European centers. Overall, LAAO procedures guided by micro- or mini-TEE were performed under MCS in 530 patients, while GA was used in 16 cases. Conversion to standard TEE and GA was required in only 4 patients (0.7%) because of suboptimal image quality. Image quality obtained with miniaturized TEE was considered adequate to guide LAAO procedures. Moreover, because these probes use imaging planes similar to those of conventional TEE, the learning curve for operators may be relatively short. However, the technique still requires an experienced echocardiographer and does not completely eliminate the risk of TEE-related esophageal injury, although this risk may be lower compared with standard TEE probes. Importantly, miniaturized TEE probes were well tolerated by the majority of patients, without significant airway complications [28]. However, attention to probe integrity, temperature monitoring, and imaging time remains important to minimize potential esophageal complications [42]. Additional studies, including propensity score–matched analyses, have supported the use of miniaturized TEE as a valid alternative imaging modality for guiding LAAO procedures [43,44] (Table 1).

### 4.3. Transesophageal–Intracardiac Echocardiography (TE-ICE)

Transesophageal intracardiac echocardiography (TE-ICE) has recently emerged as an alternative imaging strategy to guide structural heart interventions while potentially reducing the invasiveness of standard TEE [45] (Table 1). The use of an ICE catheter through the esophageal route has already been investigated in other settings, showing a favorable safety profile compared with conventional TEE, particularly for excluding LAA thrombus and for guiding transcatheter patent foramen ovale closure and selected congenital heart disease procedures [46,47,48]. However, the overall experience with TE-ICE in structural heart interventions remains limited [45,49]. In the setting of LAAO, the DIONISO study was the first to specifically evaluate the feasibility, safety, and efficacy of TE-ICE as a routine guidance modality in an all-comers population [34]. The procedure is performed under topical pharyngeal anesthesia only, typically using lidocaine spray, without the need for additional sedation. An 8-F AcuNav™ catheter (Siemens Medical Solutions USA, Inc., Malvern, PA, USA), with a diameter of approximately 2.66 mm, is advanced into the mid-esophagus as a standard TEE probe. The catheter incorporates a 64-element phased-array transducer with Doppler capabilities, operates at frequencies ranging from 5.5 to 10 MHz, provides a 90° imaging sector with a penetration depth of up to 16 cm, and can be steered in four directions (anterior, posterior, left, and right). In DIONISO, esophageal intubation with the ICE catheter was successfully achieved in all patients and was well tolerated using lidocaine spray alone. LAAO guided by TE-ICE combined with fluoroscopy proved feasible and safe, with long-term technical success consistent with that reported in large series performed under standard TEE guidance. The procedure was aborted in only one patient because of complex LAA anatomy, and conversion to conventional TEE was required in only two cases. Subsequently, a comparative study further evaluated this approach. The authors compared TEE and TE-ICE as routine imaging guidance modalities for LAAO in an all-comers population, using propensity score matching to reduce selection bias [35]. Technical success was similar between the TE-ICE and TEE groups (98% vs. 96%; OR 2.01; 95% CI: 0.28–22.76; *p* = 0.68). Of the three technical failures in the TE-ICE group, two were related to the need to switch to conventional TEE to optimize image quality. These results are consistent with previous studies and large real-world registries using TEE or trans-femoral ICE, reporting technical success rates between 90.0% and 98.3% [50,51,52,53,54]. The small size of the probe may result in suboptimal contact with the esophageal walls, making it more susceptible to even minor swallowing movements by the patient. In addition, the technique is not yet standardized; however, the images are largely comparable to those obtained with conventional TEE, making the modality familiar to cardiologists and potentially facilitating the operator learning curve. Currently, X-plane imaging is not available. A 3D probe has recently been introduced, which may help overcome this limitation [45]. Despite these promising preliminary results, TE-ICE should be considered an experimental and early-stage technique. The currently available evidence is limited and largely derived from single-center experiences, which may restrict the generalizability of the findings. In addition, important limitations related to cost, availability, and procedural standardization remain. The main clinical experience with this approach derives from a single center, where TE-ICE is currently performed within a protocol approved by the local ethics committee.

## 5. Comparative Considerations Across Imaging Modalities

Although TEE, ICE, miniaturized TEE, and TE-ICE have all demonstrated feasibility and safety for guiding LAAO, each modality presents distinct trade-offs that may influence procedural strategy (Table 2). TEE remains the reference standard due to its high image quality, multiplanar capabilities, and widespread familiarity, but its dependence on general anesthesia and esophageal instrumentation may limit its use in frail patients. ICE allows for a fully minimalist approach under local anesthesia and enables operator-controlled imaging; however, it requires additional vascular access, is associated with a learning curve, and may necessitate left atrial positioning to achieve optimal visualization. Miniaturized TEE probes reduce invasiveness while preserving familiar imaging views, although they currently lack three-dimensional capabilities and still require an echocardiographer. TE-ICE represents a promising hybrid solution that may combine reduced invasiveness with procedural familiarity, but its role is still evolving and requires further validation (Table 3). Despite the increasing number of studies supporting these approaches, several limitations should be acknowledged. Most available data derive from observational studies and single-center experiences, with a limited number of randomized or prospective comparative trials. While multicenter data are available, these are predominantly limited to ICE and miniaturized TEE, whereas evidence for TE-ICE remains confined to single-center experiences. In addition, many studies reporting comparable outcomes between ICE and TEE have been conducted in high-volume centers with experienced operators, which may limit the generalizability of these findings to real-world settings. Furthermore, ICE-guided procedures are associated with a learning curve, and different catheter positioning strategies have been described, requiring specific training and operator experience [32]. The interpretation of ICE images may also be less intuitive compared with TEE, given the different imaging perspectives. In contrast, TE-ICE provides views more closely resembling standard TEE, potentially facilitating operator familiarity, although the technique remains non-standardized and currently supported by limited single-center experience.

Overall, no single imaging modality can be considered universally superior, and each technique should be interpreted within the context of patient characteristics, anatomical complexity, and institutional expertise (Table 3).

Table 3 summarizes the main technical characteristics of the available imaging modalities. Differences in imaging capabilities (e.g., 3D and X-plane), access routes, and probe size may have relevant clinical implications in terms of operator familiarity, learning curve, and feasibility of minimalist approaches. These technical differences have relevant clinical implications. In particular, modalities providing familiar imaging views, such as TEE and TE-ICE, may facilitate operator adoption, whereas ICE requires specific expertise and is associated with a steeper learning curve. In addition, the absence of advanced imaging features, such as X-plane and 3D, may influence procedural planning in complex anatomies.

## 6. Toward a Tailored Minimalist LAAO Approach

**Figure 1:**
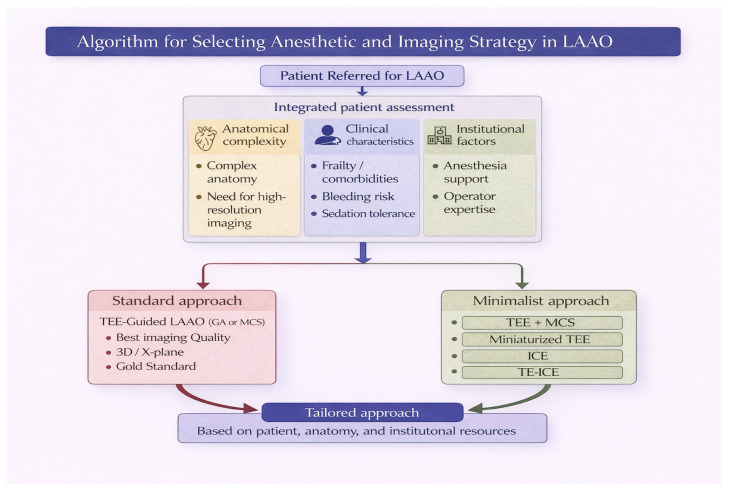
Algorithm for Selecting Anesthetic and Imaging Strategy in Left Atrial Appendage Occlusion (LAAO). Schematic representation of a decision-making pathway for selecting the most appropriate anesthetic and imaging strategy in patients undergoing LAAO. The algorithm integrates anatomical complexity, clinical characteristics (including frailty, comorbidities, bleeding risk, and sedation tolerance), and institutional factors such as availability of anesthesia support and operator expertise. Patients with high anatomical complexity or requiring high-resolution imaging are preferentially directed toward a standard approach using TEE, typically under GA or MCS. In contrast, patients with lower anatomical complexity may be considered for a minimalist approach, including TEE under MCS, miniaturized TEE, ICE, or TE-ICE. Ultimately, the selection of the procedural strategy should be individualized, based on patient characteristics, anatomical considerations, institutional resources, and operator experience. TEE = transesophageal echocardiography; GA = general anesthesia; MCS = moderate conscious sedation; ICE = intracardiac echocardiography; TE-ICE = transesophageal–intracardiac echocardiography.

The growing availability of alternative anesthetic strategies and imaging modalities for LAAO has shifted the procedural paradigm from a standardized approach toward a more individualized, patient-centered model. Rather than a one-size-fits-all strategy, the selection of an anesthetic and imaging guidance should be tailored according to patient characteristics, institutional resources, and operator expertise. Patient-related factors play a central role in this decision-making process. Frailty, comorbidities, and airway characteristics may favor minimalist approaches, avoiding GA. Conversely, more complex anatomies, high-risk transseptal puncture, or the need for optimal imaging resolution may still support the use of TEE under GA. Institutional factors are equally relevant. The availability of dedicated anesthesiology support, imaging expertise, and catheterization laboratory workflow organization may influence the feasibility and efficiency of different strategies. Operator experience represents another key determinant. Techniques such as ICE-guided LAAO are associated with a learning curve, and their safe adoption requires adequate training and procedural standardization. Structured educational programs, proctoring, and simulation-based training may facilitate this transition and expand the applicability of minimalist strategies. Importantly, current evidence suggests that different combinations of anesthetic and imaging strategies yield comparable procedural success and clinical outcomes, supporting flexibility in clinical practice. Therefore, the choice of procedural strategy should not be driven by presumed superiority of one modality over another, but rather by a balanced integration of patient needs, procedural complexity, and local expertise. In this evolving landscape, a tailored approach to LAAO may optimize procedural safety, resource utilization, and patient experience, ultimately contributing to a more sustainable and efficient model of care (Figure 1). In this context, preprocedural planning plays a key role in optimizing procedural efficiency. A comprehensive preprocedural assessment may facilitate the selection of the most appropriate anesthetic and imaging strategy, potentially reducing intraprocedural complexity and improving workflow, particularly when adopting minimalist approaches. In the context of minimalist LAAO strategies, device selection represents an additional key factor that may influence procedural feasibility and outcomes. Currently, the most widely used devices include the Watchman FLX (Boston Scientific) and the Amplatzer Amulet (Abbott), each characterized by specific design features that may be advantageous in different anatomical scenarios. The Watchman FLX, with its enhanced flexibility, closed distal end, and high conformability, allows controlled deployment, full recapture, and repositioning, which may facilitate procedural efficiency and safety, particularly in simplified procedural settings. Conversely, the Amulet device, based on a dual-seal mechanism with a lobe and disc configuration, may provide advantages in selected anatomies, especially in terms of achieving complete sealing and minimizing peri-device leak. Therefore, the choice of device should not be considered uniform but rather tailored according to multiple factors, including LAA morphology, landing zone characteristics, and ostial dimensions. In addition, institutional availability and operator experience with a specific device play a crucial role, as familiarity with device-specific implantation techniques may directly impact procedural success and complication rates. In this setting, preprocedural imaging, such as computed tomography (CT), may further support a tailored approach by providing a comprehensive assessment of LAA anatomy, including size, morphology, orientation, and relationship with surrounding structures. CT-based preplanning may improve device selection, facilitate transseptal puncture strategy, and reduce procedural uncertainty, thus contributing to a more streamlined and efficient workflow. This approach is particularly relevant in minimalist strategies, where accurate preprocedural planning may compensate for the reduced reliance on intraprocedural imaging and further enhance procedural safety and reproducibility [55,56,57].

Recent large randomized trials, including CLOSURE-AF and CHAMPION-AF, have provided important insights into the clinical effectiveness and safety of LAAO compared with medical therapy [58,59]. In the CLOSURE-AF trial, LAAO did not demonstrate non-inferiority compared with best medical therapy in a high-risk population, with a high rate of adverse events observed in both groups. Conversely, the CHAMPION-AF trial showed that LAAO was non-inferior to OAC therapy for the prevention of thromboembolic events and was associated with a significant reduction in non–procedure-related bleeding. However, these trials were not designed to specifically evaluate minimalist anesthetic or imaging strategies. The majority of periprocedural complications reported appear to be primarily device-related rather than anesthesia-driven.

In this context, speckle tracking echocardiography is emerging as a valuable non-invasive tool for the assessment of left atrial and left atrial appendage function in patients with atrial fibrillation. Recent studies have demonstrated a strong association between impaired left atrial deformation indices and left atrial appendage dysfunction, including thrombus formation [59]. These parameters may provide incremental value for thromboembolic risk stratification beyond conventional clinical scores. From a procedural perspective, speckle tracking–derived indices may support preprocedural decision-making, contributing to the identification of patients at higher thromboembolic risk and potentially guiding the selection of the most appropriate imaging and anesthetic strategy. In selected cases, this approach may help refine patient selection and reduce reliance on invasive imaging, although further prospective validation is required [60,61]. These considerations suggest that, while minimalist approaches may improve procedural efficiency, patient comfort, and resource utilization, their direct impact on major procedural complications may be limited.

## 7. Conclusions

As LAAO continues to expand, there is an increasing need for procedural strategies that reduce invasiveness while maintaining high standards of safety. Although TEE under general anesthesia remains the reference standard, alternative approaches are progressively emerging, including TEE under MCS, ICE, miniaturized TEE probes, and TE-ICE, each with specific advantages and limitations. Ongoing technological advancements and growing clinical experience are expected to progressively overcome the current limitations of each imaging modality.

Currently, no single strategy appears universally superior. Instead, the optimal approach should be tailored according to patient characteristics, anatomical complexity, institutional resources, and operator expertise. In this context, careful preprocedural planning plays a central role in guiding the selection of the most appropriate imaging and procedural strategy. Overall, a tailored minimalist approach represents a pragmatic and patient-centered model that may improve procedural efficiency, reduce resource utilization, and maintain clinical outcomes in contemporary LAAO practice. In this evolving landscape, a tailored and flexible approach to imaging guidance is essential to ensure both procedural safety and efficiency in LAAO practice.

## Figures and Tables

**Table 1:** Summary of studies on anesthetic and imaging strategies for LAAO.

Study (Year)	Design	N	Imaging	Anesthesia	Device	Technical Success (%)	Safety (%)	Conversion Rate (%)
Marmagkiolis et al., 2021 [23]	Prospective	112	TEE	MCS	Watchman	100%	100%	0%
Vizzari et al., 2025 [21]	Observational	100	TEE	MCS	Watchman FLX	100%	100%	6%
Ates et al., 2020 [20]	Prospective	112	TEE	MCS	Watchman	100%	100%	0%
Nielsen-Kudsk et al., 2022 [30]	Prospective	100	ICE	Local	Watchman FLX	100%	high	0%
Nielsen-Kudsk et al., 2019 [31]	Observational	1085 (ICE:130; TEE:955)	ICE vs. TEE	Mixes	Amulet	100%	High	0%
Berti et al., 2018 [32]	Observational	604 (ICE:187; TEE:417)	ICE vs. TEE	Mixed	Mixed	≥94%	93.5% (ICE) 95.8% (TEE)	NA
Pastormerlo et al., 2023 [33]	Observational	772 (ICE:149; TEE:623)	ICE vs. TEE	Mixed	Watchman FLX	98.7%	98.5%	NA
Aminian et al., 2023 [28]	Multicenter	546	Micro/Mini-TEE	MCS	Mixed	98%	97.1%	0.7%
Patti et al., 2019 [25]	Observational	22	Micro-TEE	MCS	Amulet	Feasible	NA	NA
DIONISO study 2024 [34]	Prospective	114	TE-ICE	Topical	Mixed	97.5%	97.4%	1.7%
Laterra et al., 2025 [35]	Comparative (PSM)	282	TE-ICE vs. TEE	Mixed	Mixed	98% vs. 96%	93% vs. 92%	~2–3%

TEE = transesophageal echocardiography; ICE = intracardiac echocardiography; TE-ICE = transesophageal–intracardiac echocardiography; MCS = moderate conscious sedation; PSM = Propensity Score Matching; VS = Versus; NA = Not Available.

**Table 2:** Comparison of Imaging Techniques for Guidance of Left Atrial Appendage Occlusion.

Modality	Anesthesia	Invasiveness	Image Quality	Learning Curve	Advantages	Limitations
TEE	GA/ MCS	Semi-invasive	Excellent	Moderate	Gold standard, 3D, X-plane	GA, esophageal risks
ICE (TF)	Local	Invasive (vascular)	Good	Steep	No GA, operator control	Cost, access, quality of images, No X-plane
TE-ICE	Topical	Minimally invasive	Good	Moderate	No GA, familiar views	No X-plane, Cost
Micro/mini-TEE	MCS	Minimally invasive	Moderate	Moderate	No GA, familiar views	No 3D

TEE = transesophageal echocardiography; ICE = intracardiac echocardiography; TE-ICE = transesophageal–intracardiac echocardiography; MCS = moderate conscious sedation; GA = general anesthesia; TF = transfemoral; 3D = three-dimensional.

**Table 3:** Technical characteristics of imaging tools.

Parameter	TEE	ICE	TE-ICE	MicroTEE
Probe size	~10 mm	8F (2.6 mm)	8F (2.6 mm)	5–7 mm
3D imaging	Yes	Limited (new)	Limited (new)	No
X-plane	Yes	No	No	No
Doppler	Yes	Yes	Yes	Yes
Access	Esophageal	Venous	Esophageal	Esophageal

TEE = transesophageal echocardiography; ICE = intracardiac echocardiography; TE-ICE = transesophageal–intracardiac echocardiography; 3D = three-dimensional; F = French.

## Data Availability

No new data were created or analyzed in this study.

## Abbreviations

The following abbreviations are used in this manuscript:

AF	Atrial Fibrillation
GA	General Anesthesia
ICE	Intracardiac Echocardiography
LAA	Left Atrial Appendage
LAAO	Left Atrial Appendage Occlusion
MCS	Moderate Conscious Sedation
TE-ICE	Transesophageal–intracardiac Echocardiography
TEE	Transesophageal Echocardiography
